# Intrasaccular Treatment of Intracranial Aneurysms: A Comprehensive Review

**DOI:** 10.3390/jcm13206162

**Published:** 2024-10-16

**Authors:** Miriam M. Shao, Timothy G. White, Jared B. Bassett, Ehsan Dowlati, Shyle H. Mehta, Cassidy Werner, Danielle Golub, Kevin A. Shah, Amir R. Dehdashti, Ina Teron, Thomas Link, Athos Patsalides, Henry H. Woo

**Affiliations:** Department of Neurosurgery, Donald and Barbara Zucker School of Medicine at Hofstra/Northwell, North Shore University Hospital, Manhasset, NY 11030, USAsmehta13@northwell.edu (S.H.M.);

**Keywords:** intrasaccular flow disruption, Woven EndoBridge device (WEB), LUNA/Artisse Embolization Device, Contour, Saccular Endovascular Aneurysm Lattice Embolization System (SEAL), Medina Embolization Device (MED), Trenza

## Abstract

**Background**: The endovascular treatment of complex intracranial aneurysms, such as wide-neck aneurysms (WNAs), remains a challenge. More established endovascular techniques, which include balloon-assisted coiling, stent-assisted coiling, and flow diversion, all have their drawbacks. Intrasaccular flow disruptor devices have emerged as a useful tool for the neurointerventionalist. **Methods**: Here, we discuss landmark studies and provide a comprehensive, narrative review of the Woven EndoBridge (WEB; Microvention, Alisa Viejo, CA, USA), Artisse (Medtronic, Irvine, CA, USA), Contour (Stryker, Kalamazoo, MI, USA), Saccular Endovascular Aneurysm Lattice Embolization System (SEAL; Galaxy Therapeutics Inc, Milpitas, CA, USA), Medina (Medtronic, Irvine, CA, USA), and Trenza (Stryker, Kalamazoo, MI, USA) devices. **Results**: Intrasaccular devices have proven to be effective in treating complex aneurysms like WNAs. **Conclusions**: Intrasaccular flow disruptors have emerged as a new class of effective endovascular therapy, and results of ongoing clinical studies for the newer devices (e.g., SEAL and Trenza) are much anticipated.

## 1. Introduction

Endovascular techniques like coiling have become a preferred treatment in the management of many intracranial aneurysms, but coiling has been associated with both decreased occlusion rates as well as increased procedural risk when used to treat wide-neck aneurysms (WNAs) [1]. WNAs are commonly defined as having a neck diameter of ≥4 mm or a dome-to-neck ratio of <2.0 [2]. Wide-neck bifurcation (WNBAs) aneurysms occur at sites where there are two branching vessels such as the basilar apex, internal carotid artery (ICA) terminus, middle cerebral artery (MCA) bifurcation, and A1-2 junction [2].

Whereas the neck of a narrow-neck saccular aneurysm can confine coils within the dome, WNAs and WNBAs pose a challenge for conventional endovascular coiling because of the increased risk of coil protrusion into the parent vessel. In addition, there is a high rate of aneurysm recurrence reported in the literature, which may be related to increased blood flow across the neck promoting coil compaction [3]. Given the difficulty of treating WNBAs with traditional endovascular techniques, treatment modalities have evolved to include alternative mechanisms for treating these aneurysms. These techniques include balloon-assisted coiling (BAC), stent-assisted coiling (SAC), and flow diversion (FD) [2,4]. While endoluminal remodeling using FD or neck bridging stents has been widely used and described, the deployment of an endoluminal device may be anatomically challenging and generally involves jailing one of the branches of the bifurcation, which may be associated with significant morbidity and potentially suboptimal occlusion [5]. Furthermore, placement of endoluminal devices can be challenging in the ruptured setting given the need for dual antiplatelet therapy (DAPT) [6]. 

Intrasaccular flow disruptors were developed to treat WNBAs and overcome the shortcomings of the aforementioned treatment modalities. Similar to endoluminal FD, intrasaccular devices rely on altering intra-aneurysm hemodynamics and promoting thrombosis of the aneurysm while providing a high degree of metal coverage at the neck of the aneurysm as a scaffold to promote endothelization. Currently, the following six devices are being tested and used worldwide: Woven EndoBridge (WEB; Microvention, Alisa Viejo, CA, USA), Artisse (Medtronic, Irvine, CA, USA), Contour (Stryker, Kalamazoo, MI, USA), Saccular Endovascular Aneurysm Lattice Embolization System (SEAL; Galaxy Therapeutics Inc, Milpitas, CA, USA), Medina (Medtronic, Irvine, CA, USA), and Trenza (Stryker, Kalamazoo, MI, USA). Here, we provide a comprehensive, narrative review of these intrasaccular flow disruptor devices.

### Endovascular Treatment Options for WNBAs

WNBAs pose particular challenges that make them less amenable to conventional endovascular coiling. Vessel branch points favor aneurysm formation and eventual recurrence after treatment because of turbulent blood flow [7]. The placement of an endoluminal device to promote aneurysm obliteration risks ischemia to a jailed branching vessel or in-stent-stenosis to a stented vessel, both of which can lead to stroke. Therefore, microsurgical clipping has historically been the standard treatment approach for WNAs. In one-year follow-up results of a post hoc analysis of the Barrow Ruptured Aneurysm Trial (BRAT) and of the Endovascular Therapy Versus Microsurgical Clipping of Ruptured Wide Neck Aneurysms (EVERRUN) registry, Mascitelli et al. showed that endovascular therapy and microsurgical therapy produced similar clinical outcomes. However, microsurgical clipping yielded higher rates of complete and adequate aneurysm occlusion [8,9,10]. In particular, MCA WNAs—because of their distal location and frequent incorporation of narrow vessels—are still considered heavily for microsurgical clipping over endovascular therapy [8,11].

BAC, in which a balloon catheter is inflated within the parent vessel prior to or during the deployment of coils, is often used for WNBA treatment. The balloon effectively covers the neck of the aneurysm, thereby allowing the coils to frame appropriately within the aneurysm sac. This facilitates a stable conglomerate coil mass and increased packing density [12,13]. This also improves aneurysm occlusion and reduces the risk of aneurysm recanalization while preventing coil mass herniation into the parent vessel [2,14]. In the setting of bifurcation aneurysms, previous authors have described using two balloon microcatheters and implementing the kissing balloon technique, in which two balloons are inflated simultaneously in the two bifurcation branches [2,15]. 

With SAC, a stent is deployed across the neck of the aneurysm prior to or after coil deployment. A microcatheter can be navigated through the stent or jailed into the aneurysm using a two-catheter technique. Many structural stents can also be placed through the dual lumen balloon microcatheters, allowing for combination balloon- and stent-assisted coiling. However, a single device may not be adequate to remodel a bifurcation and maintain the patency of both vessels. It is possible to deploy complex stent constructs such as Y, X, or T stent constructs to maintain parent vessels and increase aneurysm occlusion [9]. 

BAC and SAC can also be performed using transcirculation catheterization techniques. Transcirculation catheterization involves navigating a catheter, balloon, or stent delivery device either from one arterial side to the contralateral side or from the anterior to posterior circulation [16]. The target lesion can be accessed by crossing a communicating vessel—such as the anterior communicating artery, posterior communicating artery, and vertebral arteries—rather than the parent vessel [17]. Previous studies have shown transcirculation catheterization to be effective in treating carotid terminus aneurysms, those involving a fetal posterior cerebral artery, and WNBAs involving the basilar tip with P1-P2 stenting using the posterior communicating artery [18]. It is important to note that the transcirculation technique requires two sites of access and potential navigation of small communicating arteries in the circle of Willis with microcatheterization [16,18]. 

However, these described adjunctive methods increase procedure complexity and can be technically challenging. In addition, SAC requires the use of DAPT to reduce stent thrombogenicity. SAC is therefore not favored for use in the setting of ruptured aneurysms [2,19,20]. Furthermore, Fiorella et al. conducted a meta-analysis in 2017 and showed that the complete occlusion rate for WNBAs increased only modestly to 59.4% with SAC from 46.3% with conventional coiling [21].

FD uses high metal surface area braided stents to limit aneurysm inflow, thus promoting intra-aneurysm thrombosis and neck bridging endothelialization [2,22]. While FD has taken over as the treatment of choice for many sidewall aneurysms, it has failed to demonstrate the same efficacy when it comes to bifurcation aneurysms. The use of FD for WNBAs remains controversial, as it is associated with lower occlusion rates and higher morbidity rates [4]. Furthermore, the deployment of the endoluminal device for FD involves risks similar to those of SAC [5]. While in some cases, the covered vessel may adaptively change over time, the jailing of large branching vessels has been associated with ischemic complications [5]. While newer generation devices offer the advantage of surface modification to alter their thrombogenicity, level 1 evidence is yet to support antiplatelet monotherapy [23].

Studies evaluating FD specifically for WNBAs have produced mixed results. Gawlitza et al. reported ischemic perforator lesions in 41.2% of WNBAs involving the anterior circulation with nineteen cortical branches that were “jailed” by FD. In addition, 17.6% of patients had transient symptomatic ischemic events in perforator territories that resolved within 24 h, and 29.4% were found to have asymptomatic lacunar infarcts on follow-up MRI. Overall, 33.3% of the aneurysms were completely occluded at an average follow-up of 7.9 months [24]. Saleme et al. also prospectively evaluated FD for thirty-seven WNBAs of the anterior circulation in thirty-two patients. At the eighteen-month follow-up, 97.3% of the aneurysms had complete occlusion. In addition, 9.4% of the patients had a new permanent neurological deficit, and WNBAs with branches that did not have direct collateral supply (i.e., MCA bifurcation aneurysms) had increased risk of ischemic events compared with WNBAs with branches that had collateral supply (anterior communicating artery and ICA bifurcation aneurysms) [25].

Multicenter studies and systematic reviews echo the mixed results associated with FD for WNBAs. Salem et al. and Diestro et al. analyzed the use of FD in MCA bifurcation aneurysms. Both displayed an adequate occlusion rate of approximately 80% with a follow-up time of approximately one year, and both showed low rates (less than 2% of cases) of hemorrhagic complications. However, thromboembolic events occurred more frequently. Salem et al. reported ischemic complications in 8% of cases with symptomatic and permanent complications encountered in 5.7% and 2.3% of patients, respectively. Of note, occlusion of jailed branches was found in 11.5% of cases, with symptomatic sequelae in 2.3% of these cases [26]. Diestro et al. reported thromboembolic events in as many as 16.7% of cases, although only 5.6% of patients had permanent deficits [27]. Kashkoush et al. conducted a systematic review of nineteen studies. Basilar tip aneurysms were included in addition to anterior circulation WNBAs. At a follow-up period of sixteen months, complete occlusion occurred in 69% of the aneurysms, but the complication rate was as high as 22%. Ischemic events comprised the majority of complications at 16%. The main causes of ischemic events were jailed branch hypoperfusion (47%) and in-stent thrombosis (38%). In addition, 7% of patients had permanent symptomatic complications [5]. 

Many other devices have been tried to assist in the embolization of these challenging lesions; their uses have been reviewed elsewhere. Other alternative treatment techniques have included devices like PulseRider (Cerenovus, Johnson & Johnson, New Brunswick, NJ, USA), eClips (EVasc Medical System, Vancouver, BC, Canada), pCONUS (WallabyPhenox, Bochum, Germany), Nautilus (Endostream Medical, Tel Aviv, Israel), and Neqstent (Stryker, Kalamazoo, MI, USA) [28,29,30,31,32,33,34,35,36,37,38,39,40,41]. Each of these devices attempts to reconstruct the aneurysm neck or aneurysm parent vessel interface to improve coiling and provide some degree of neck metal coverage to enhance endothelialization and aneurysm closure. Ultimately, while some of the newer devices have shown promise in establishing an aneurysm base to improve coil packing, no one device has taken over as a reliable alternative.

## 2. Intrasaccular Devices

### 2.1. Intrasaccular Devics Overview

Intrasaccular devices have recently emerged for the treatment of more complex aneurysms, initially with the indication for WNBAs. Intrasaccular devices occupy the neck of the aneurysm, with high metal coverage at the ostium of the aneurysm. Their mechanism of action involves disrupting blood flow at the interface between the aneurysm neck and parent vessel for the purpose of inducing thrombosis and aneurysm occlusion [42]. Intrasaccular devices do not involve any endoluminal constructs to reconstruct the parent vessel. However, a downside of intrasaccular devices compared with endoluminal therapies—specifically flow diversion—is the need for aneurysm catheterization, which has been shown to pose an increased risk of intraoperative aneurysm rupture due to sac manipulation [43]. Nonetheless, given their lack of endoluminal metal within parent vessels, intrasaccular devices do not inherently require DAPT if device protrusion is not encountered, and bail-out stenting is not required [44]. 

**Figure 1:**
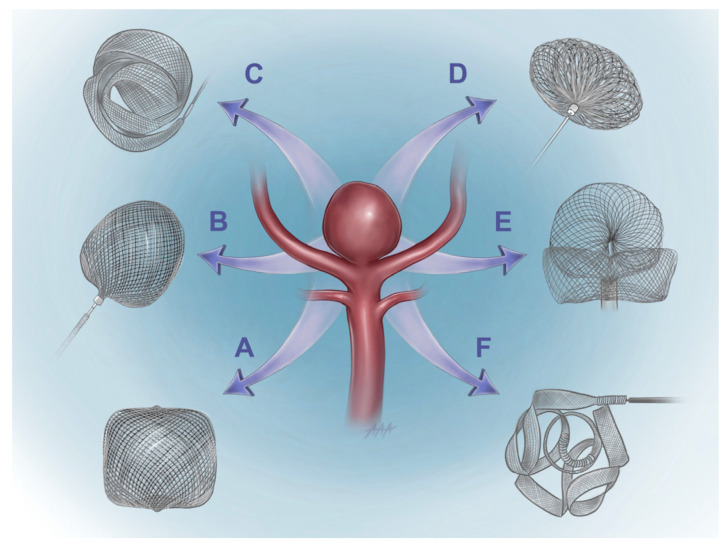
Illustration of intrasaccular flow disruptor devices for the treatment of WBAs and WNBAs. A—WEB. B—LUNA/Artisse. C—MED. D—Contour. E—SEAL. The illustrated SEAL device is the configuration that includes both the ovoid upper loop and base bridging component. F—Trenza.

The first intrasaccular device to be widely used was the Woven EndoBridge (WEB) device, followed by the LUNA/Artisse Embolization System. Both devices were introduced in 2010. WEB gained CE Mark and FDA approval in 2010 and December 2018, respectively; LUNA/Artisse gained CE Mark approval in 2011. The Medina Embolization Device (MED) was the next device to be introduced, receiving CE Mark approval in 2014; the first clinical experience with MED was reported in 2015 [45]. The first reports of the use of Contour and SEAL were in 2020 and 2021, respectively [4,5,6,7,8,9,10,11,12,13,14,15,16,17,18,19,20,21,22,23,24,25,26,27,28,29,30,31,32,33,34,35,36,37,38,39,40,41,42,43,44,45,46,47,48,49]. Finally, Trenza was recently introduced to clinical practice with only one series reported in the literature in 2024. Contour gained CE Mark approval in 2020, but SEAL and Trenza have yet to receive either CE Mark or FDA approval. Figure 1 provides an illustration of these six intrasaccular flow disruptor devices.

### 2.2. Woven EndoBridge (WEB) Device

The WEB device acts as a self-expanding mesh cage when it is deployed and electrolytically detached at the aneurysm neck. The newest generation is made of a nitinol-drawn filled tube (DFT) with platinum core wires for improved visibility. The implant conforms to the aneurysm wall and relies on lateral compression to maintain its shape and prevent device compression [48,50]. It also provides full aneurysm neck coverage with its high-density mesh and functions as an “intrasaccular flow disruptor” [44]. The original version (WEB-DL) was a dual layer with up to 288 braided wires, but since 2013, the newer versions (WEB-SL and WEB-SLS) consist of only a single layer with between 114 and 216 wires. The original WEB-SL is cylindrical in shape, while the WEB-SLS is spherical in shape. The transition to a single-layer construction enabled a better balance in radial force upon deployment, porosity, and thrombogenicity [48]. Recently, the WEB 17 device was introduced as the latest advancement with a reduction in the number of wires to between 72 and 108, facilitating device deliverability through a 0.017-inch microcatheter and treatment of smaller aneurysms. Spelle et al. demonstrated in the CLinical EValuation of WEB 17 device in intracranial aneuRysms (CLEVER) study that this miniaturization represents an improvement in its ease of use [51,52]. 

In the traditional WEB sizing method, 1 to 2 mm is added to the average width of the aneurysm, and 1 to 2 mm is subtracted from the height of the aneurysm [50]. Height is subtracted because of the expected elongation of the device along the vertical axis of the aneurysm from lateral compression. Many have instead suggested matching WEB device volume to aneurysm volume for optimal device sizing; however, this technique is not possible when using the technique of “corking the neck” of irregularly shaped aneurysms [50,53]. In this technique, the WEB device is still positioned at the aneurysm neck in a way that allows it to block blood inflow into the aneurysm completely. However, it does not need to be parked in a way that the top of the device abuts the top of the aneurysm dome [53]. WEB-SL comes in sizes ranging from 4 mm × 2 mm to 11 mm × 9 mm, and WEB-SLS comes in sizes ranging from 4 mm to 11 mm. Regarding the WEB 17 series, the WEB 17 SL comes in sizes ranging from 2 mm × 3 mm to 7 mm × 4 mm, and the WEB 17 SLS comes in diameters ranging from 4 mm to 7 mm [54]. The WEB device is delivered through a 0.017- to 0.033-inch diameter microcatheter depending on the size chosen, and it is fully resheathable after delivery [42].

The “French Observatory study” and the following WEBCAST and WEBCAST 2 studies conducted in Europe were the first multicenter, prospective studies to evaluate WEB in patients [55,56,57]. Collectively, these three studies enrolled 168 patients with 169 aneurysms and demonstrated the safety and efficacy of the WEB device. At the one-year follow-up, there was complete aneurysm occlusion in 52.9% of cases, neck remnant in 26.1%, and aneurysm remnant in 20.9%. Overall, 6.9% required retreatment [58]. At the five-year follow-up, 100 patients remained in the study, and there was complete occlusion or neck remnant in 77.9% of aneurysms. Retreatment was performed for 11.6% of aneurysms and mostly occurred during the first two years post-treatment. The mortality rate at the five-year follow-up was 7%; however, only 1% of the mortality rate was related to the procedure (retroperitoneal hematoma), and there was no mortality related to the WEB device. There were also no adverse events related to the WEB device [59].

The WEB-IT study was a North American multicenter, prospective study with 150 patients. Adequate occlusion was defined as either complete occlusion or neck remnant. At the one-year follow-up, adequate occlusion was seen in 84.6%, and complete occlusion was seen in 53.8%. There were no periprocedural mortalities, and one patient had a delayed ipsilateral intraparenchymal hemorrhage on postoperative day 22. Furthermore, no patients developed significant (>50%) parent artery stenosis [60]. The results of the study demonstrated the feasibility and safety of the WEB device in the North American market and played a large role in the FDA premarket approval of WEB in 2019 for the treatment of WNBAs. At the five-year follow-up, 83 out of 150 (55.3%) patients remained in the study, with 58.1% having complete occlusion and 87.2% having adequate occlusion. Overall, 76.8% of patients had aneurysms that remained stable or had progressive occlusion compared with their one-year follow-ups. Retreatment was performed in 15.5% of patients. The mortality rate was 4.7% but with no deaths or adverse events related to the WEB device [61]. Recent meta-analyses have also confirmed adequate occlusion rates between 80% and 90% for the WEB device within the one-year follow-up [62,63,64,65]. Rooij et al. differed from most other recent meta-analyses by focusing only on WEB-SL/SLS (rather than also including WEB-DL). They presented an overall adequate occlusion rate of 83.3%, a retreatment rate of 8.4%, and a thromboembolic event rate of 5.6% [48,66]. 

Given its lack of requirement for DAPT, WEB has also been studied specifically for the treatment of ruptured aneurysms. The CLinical Assessment of WEB device in Ruptured aneurYSms (CLARYS) study was a European prospective study that included 60 patients with ruptured aneurysms. It showed that the rebleeding rate with WEB was 0% at both the one-month and one-year follow-ups. At the one-year follow-up, adequate occlusion was achieved in 87% of cases and complete occlusion in 41.3% [67]. The mortality rate at the one-month and one-year follow-ups were 1.7% and 3.8%, respectively. The morbidity, as defined by a modified Rankin Scale (mRS) > 2, at the one-month and one-year follow-ups was 15% and 9.6%, respectively. However, mortality and morbidity rates related to the WEB device were 0% [68]. These results are echoed by systematic reviews by Monteiro et al., Crinnion et al., Xie et al., and Harker et al., which studied the use of WEB in the acute phase of ruptured aneurysms with a follow-up period between four and twelve months. Adequate occlusion rates were generally found to be between 84.8% and 91% [69,70,71], with Harker et al. reporting a complete occlusion rate of 62% at only the four-month follow-up [48,72]. Overall rebleeding rates were between 1.2% and 3%, with thromboembolic rates being between 4.5% and 17%. A good clinical outcome, as evidenced by a mRS < 2, occurred in between 62.2% and 88.7% of patients [48,69,70,71,72].

While intrasaccular flow disruptors fundamentally do not require antiplatelet therapy, it is still commonplace for many neurointerventionalists to use some form of antiplatelet therapy with either DAPT or single antiplatelet therapy (SAPT). Reasons for antiplatelet use include cases of bail-out stenting, device protrusion, or proceduralist preference. The common use of antiplatelets for intrasaccular flow disruptors is seen in the results of monumental WEB studies, such as with the WEB-IT study [60,61]—as well as with the pivotal European studies (i.e., the French Observatory, WEBCAST, WEBCAST 2) [55,56,57] and Pierot’s collective analysis of European studies [58]. All had significant proportions of patients on antiplatelet therapy at both the time of procedure (WEB-IT, DAPT 69.3% and SAPT 27.3%; Pierot’s collective analysis, DAPT 45.2% and SAPT 36.9%) and follow-up (WEB-IT, 30 days DAPT 31.3% and SAPT 56.0%, 6 months DAPT 11.3% and 62.7% SAPT; Pierot’s collective analysis, 30 days DAPT 23.4% and SAPT 54.5%) [55,56,57,58]. Antiplatelets were also commonly used in the CLARYS trial that prospectively investigated WEB specifically for ruptured WNBAs. Despite 93% of the patients reporting no antiplatelet use prior to intervention, 43% of them were started on either SAPT or DAPT at the time of intervention, and 30% remained on antiplatelet therapy at the 1-month follow-up [68]. Despite the heterogeneous use of antiplatelets for WEB in practice, it is important to note that intrasaccular flow disruptors are by design extra-luminal, thereby making the use of antiplatelets not entirely necessary.

### 2.3. Artisse Embolization Device (Previously LUNA)

Similar to WEB, the Artisse Embolization Device (previously LUNA), is a self-expanding braided mesh device made of nitinol with platinum core wires and radio-opaque markers. LUNA was the earlier version of the device that had an ovoid shape; the Artisse Embolization Device is the later version that comes in two different shapes as follows: a spheroid shape or a flared, acorn-like shape. The sizes range from 4.5 to 8.0 mm in diameter. The device is delivered through a 0.021-inch microcatheter and is detached electrolytically [42]. When compared with the WEB device, the Artisse/LUNA has an atraumatic distal finished end with no distal radiopaque marker, thereby potentially decreasing the theoretical risk of rupturing the aneurysm on deployment. The largest clinical series on LUNA was conducted in Europe. It was a prospective, multicenter study that enrolled 63 patients with 64 aneurysms. Unlike the WEB device studies, however, the study included a relatively large proportion (23.4%) of sidewall, non-WNBAs. Adequate occlusion was achieved in 78.0% and 79.2% of the aneurysms at the 12-month and 36-month follow-ups, respectively. In contrast with WEB, LUNA did not exhibit device compression at follow-up that required retreatment, and the four retreatment cases were due to a poor deployment technique or device migration likely secondary to improper device sizing. The retreatment rate was 6.3% at 12 months and 4.8% at 36 months. Prior to the 12-month follow-up, there were two (3.2%) major strokes, one (1.6%) minor stroke, and three incidents of intracranial hemorrhage (ICH) in two patients, with overall rates of thromboembolic events, morbidity, and mortality comparable to those seen with the WEB device. In both ICH patients, procedural hemorrhage was due to aneurysm perforation by the microcatheter. One of the patients also experienced spontaneous rupture of a contralateral, untreated aneurysm at post-procedure day 71. One additional procedure involved the rupture of a cavernous carotid aneurysm and iatrogenic creation of a carotid-cavernous fistula; however, there was no associated ICH. There was one case of mortality and one case of morbidity based on mRS at the 36-month follow-up [73]. Most aneurysms treated were smaller than 10mm in diameter and unruptured. The only publication utilizing the Artisse device reported nine patients with modest results. They found a 66.7% adequate occlusion followed by 57.1% adequate occlusion at six and thirty-six months, respectively, with two significant procedural complications [74]. It remains to be seen how the long-term effectiveness and popularity of this device compare to the WEB device. 

### 2.4. Medina Embolization Device (MED)

The Medina embolization device (MED) combines the design of detachable coils and intrasaccular flow disruptors. It is a three-dimensional device with braided filaments oriented along the loops of the core wire to increase neck coverage. Similar to WEB and Artisse, the self-expanding mesh provides flow diversion at the neck while anchoring to the wall of the aneurysm. The sizing of the device follows the same principles of sizing with coils. Additionally, as with coils, the device can be recaptured in the microcatheter and repositioned. 

In vitro and in vivo studies in animals have yielded important information regarding the MED. Frolich et al. constructed ten vascular flow models based on ten different patients’ various ICA aneurysm morphologies. Using high-frequency digital subtraction angiography to analyze aneurysmal flow velocity and flat detector computed tomography, they showed that higher neck coverage by the MED and smaller aneurysmal neck size significantly correlated with greater intra-aneurysmal flow disruption (as indicated by flow velocity reduction) by the MED [75]. This phenomenon was attributed to increased braid compaction across the neck, which increased metal coverage and consequently decreased inflow, highlighting the importance of intrasaccular device configuration across the neck. In an in vivo study using lateral wall carotid aneurysms in canines, Fahed et al. compared using the MED only, the MED with coils, and coils only. At 3 months, angiographic occlusion rates were similar across all three groups (68.7% vs. 56.2% vs. 50.0%) [76].

In clinical studies, multiple feasibility and safety studies have shown promise. Sourour et al. demonstrated successful deployment in 13 cases. Specifically, they aimed to use the MED as a frame and fill the rest of the aneurysm with further MED fillers or coils. To achieve optimal positioning and neck coverage with the petals, they attempted an average of 2.5 deployments. They noted a complete occlusion rate of 83% (10/12) at the six-month follow-up angiogram, although there were two cases of recanalization at a later follow-up [77]. Another similar single-center study demonstrated efficacy in 15 patients with unruptured aneurysms. Only eleven patients had angiographic follow-up, and there was complete occlusion in four patients, stable neck remnant in six, and recanalization in one patient. In 10 of the 15 patients, adjunctive devices such as coils and endoluminal flow diverters were used. No complications were attributed to the MED [78]. Haffaf et al. reported long-term angiographic follow-up at 18 months for 20 aneurysms. Two aneurysms were unable to be treated with the MED, and there was one thromboembolic complication. Complete aneurysm occlusion was seen in 11/18 (61%) aneurysms at the end of the procedure with progressive occlusion seen at 18 months (12/15, 80%) [79]. In a study from the Karolinksa Institute, Bhogal et al. also demonstrated the safety of the device in 13 patients. However, unlike the prior studies where adjunctive coils were used in most cases, only MEDs were used in their study. The authors attributed the failures of occlusion to using only one MED or lack of coverage of the neck by the MED petals, as noted by Frolich et al. in their in vitro study [80]. Although clinical reports demonstrate efficacy and low complication rates, the inability to predict and guarantee neck coverage easily when deploying the MED is a disadvantage when compared with the WEB or Artisse/LUNA devices. The MED is currently off the market, most likely because of the inability of standalone treatment to demonstrate superior occlusion rates, as well as the costs and risks associated with the need for adjunctive devices.

### 2.5. Contour Neurovascular System

The Contour Neurovascular System is a dual-layered, radio-opaque mesh device made of nitinol DFT with platinum radio-opaque markers. In contrast to the WEB device, Contour forms a bowl-shaped hemi-sphere and conforms to the aneurysm neck when deployed. The device ranges in size to conform to aneurysm necks from 2 to 10.5 mm, allowing the treatment of smaller aneurysms than most intrasaccular devices. It is delivered through either a 0.021- or 0.027-inch microcatheter [42], unlike the smallest WEB devices, which are recommended to be delivered through a 0.017-inch microcatheter. The device is fully resheathable and can be deployed away from the aneurysm dome, thereby theoretically minimizing aneurysm manipulation. It relies on a complete neck seal and apposition to achieve adequate flow diversion. There have been multiple early single-center series published along with a limited number of multicenter cohorts leading to some early clinical data. 

Akhunbay-Fudge et al. conducted a prospective study that enrolled 11 patients at their single center in the United Kingdom. At the one-year follow-up, 55.6% had complete occlusion, and 44.4% had neck remnants. All patients had mRS < 2; however, two patients had thromboembolic events but without clinical sequelae [46]. Biondi et al. evaluated the Contour Neurovascular System on 60 unruptured aneurysms in 53 patients. At the one-year follow-up, 89.3% of the aneurysms had adequate occlusion, with 46.4% having complete occlusion. Overall, 6.7% of the aneurysms (4/60) had thromboembolic events, but only one of these events caused transient morbidity. There was no retreatment required, and there was no permanent morbidity or mortality [81]. CERUS was a prospective, multicenter study in Europe that enrolled 34 patients with 34 aneurysms. The device was successfully deployed in 32 aneurysms. At the one-year follow-up, there was complete occlusion in 69% of the aneurysms and adequate occlusion in 84%. Thromboembolic events occurred in four patients without any disabling sequelae [82]. 

More recently, Griessenauer et al. published a multicenter experience from 10 European centers evaluating the safety and efficacy of Contour embolization in 279 aneurysms—the largest Contour experience published to date. In this series, the majority of the aneurysms treated were anterior circulation aneurysms (26.5% MCA bifurcation and 26.2% anterior communicating artery complex), followed by 23.3% at the basilar tip. Additionally, 11.1% of the aneurysms were treated with Contour in the setting of acute rupture and subarachnoid hemorrhage. Overall, the authors observed a 91.5% adequate aneurysm occlusion rate at a median 12 months of follow-up with a 6.8% thromboembolic complication rate. There was no influence of rupture status on occlusion outcomes or on the rate of thromboembolic or hemorrhagic complications [83]. Multivariate analysis suggested that increasing aneurysm height tended to lower the rate of adequate occlusion. As this device conforms to the aneurysm neck instead of the dome, greater aneurysm height may result in decreased efficacy because of the lack of total aneurysmal involvement.

Hecker et al. conducted an institution comparison between Contour and WEB for the treatment of WNBAs. A total of 40 aneurysms from 34 patients were treated with Contour, and 30 aneurysms from 30 patients were treated with WEB. The median follow-up was 12 months, and only patients with at least 3 months of follow-up were included. The adequate occlusion rate was 90% for both Contour and WEB. Contour’s complete occlusion rate was 75%, and WEB’s was 63.3%. Contour had a significantly higher calculated probability of reaching complete occlusion over time. Furthermore, Contour had a significantly lower retreatment rate of 2.5% when compared with WEB’s 20%. Contour also had a significantly lower median duration of deployment at 57.5 min compared with WEB’s 75.5 min. With Contour, there was one symptomatic thromboembolism, but symptoms resolved at discharge. With WEB, there were two asymptomatic thromboembolic events and one major ischemic event [84]. Altogether, these studies display the feasibility of using Contour for WNBAs.

Overall, the Contour device offers a promising option for the treatment of WNAs; however, more data are needed. The U.S. investigational device exemption (IDE) study for Contour is actively recruiting, and like WEB-IT, the results of this trial will add to our ongoing understanding of this device’s safety and efficacy. While its use may be simpler than the other intrasaccular devices or have an easier learning curve, data on final occlusion status are needed to establish its use in clinical practice.

### 2.6. Saccular Endovascular Aneurysm Embolization System (SEAL) Device

The SEAL device is a dual-layered mesh device made of nitinol and platinum. It comes in the following configurations: one configuration is an ovoid upper loop with a base bridging component, and the other configuration includes only the base bridging component. This device also has the largest diameters when compared with the other intrasaccular devices with 20 mm sizes suitable for large aneurysms. Zoppo et al. found 80% aneurysm occlusion in a rabbit elastase model of aneurysm formation, which was associated with 100% complete histological aneurysm occlusion. While angiography demonstrated a remnant aneurysm, there was neointimal tissue ingrowth across the neck of the aneurysm. Given prior evidence suggesting improved occlusion with the dual-layered WEB device in animal models, the authors posited that there is beneficial flow disruption in dual-layered devices [85]. 

Pabon et al. presented the first SEAL device utilization in a patient. The patient presented with a ruptured, trilobed left MCA aneurysm. The aneurysm had a height of 3.46 mm, a maximum diameter of 8.47 mm, and a neck size of 4.7 mm. The SEAL device was deployed with a Headway (Microvention, Alisa Viejo, CA, USA) 0.027-inch microcatheter. At the twelve-month follow-up, the aneurysm was completely occluded, and the patient had an mRS of 0 [47]. Early results from the PRE-SEAL IT trial that have been presented at multiple conferences are encouraging, although there has been no formal publication to date. For the 33 total treated aneurysms, the 3-month follow-up showed 74.1% complete occlusion and 81.5% adequate occlusion [47,86].

### 2.7. Trenza Embolization Device

The Trenza embolization device is an intrasaccular braided frame coil implant with flow-disruption properties. It serves as a stable omega-shaped basket construct in the aneurysm that is then filled with coils. It is available to treat 6 to 12 mm WNBAs and sidewall aneurysms. The device sizing is based on the average diameter of the aneurysm in three planes, with no consideration of neck size. The device is delivered with an 8Fr distal access catheter and Excelsior 1018 (Stryker, Kalamazoo, MI, USA) microcatheter. Since the Trenza device is stiffer than normal framing coils, good proximal catheter support is needed to avoid recoil kickback of the microcatheter. In theory, Trenza’s properties of both intrasaccular flow disruption and standard coiling would lead to higher occlusion rates compared with coiling alone and to fewer thromboembolic complications compared with SAC [87].

Raj et al. shared their experience with Trenza in twelve patients with WNBAs and sidewall aneurysms of both the anterior and posterior circulation. At a median follow-up period of 6.5 months, the adequate occlusion rate was 83%. Three of the patients (25%) experienced major ischemic events, and two of the patients (17%) had permanent neurologic deficits. Both these ischemic events occurred in basilar tip aneurysm cases in which there was excessively dense coiling at the base of the aneurysm. Of note, excluding the two patients who presented with ruptured aneurysms, all patients were premedicated with antiplatelet therapy (nine with DAPT and one with only prasugrel due to an Aspirin allergy). All patients then received antiplatelet therapy for 3 months post-treatment. The prevalent use of antiplatelet therapy reflected the overall general practice of still using antiplatelets with intrasaccular devices in case of bail-out stent placement. The authors identified relatively high metal coverage at the aneurysm neck as a reason for continuing antiplatelet therapy post-treatment [87]. Future studies should confirm Trenza’s utility without supplemental antiplatelet therapy. All six devices are compared to each other in Table 1. Important studies of each of the devices are listed in Table 2.

## 3. Future Directions

The US IDE Study of the Contour NEurovasCular System for IntraCranial Aneurysm Repair (NECC) taking place in the United States, the Pre-SEAL IT study taking place in Colombia, and the Trenza Embolization Device for Intrasaccular Aneurysm Treatment study (TREAT) taking place in Finland are prospective studies that are currently underway to evaluate Contour, SEAL, and Trenza, respectively. As WEB is currently the only device approved for use in the U.S., all other intrasaccular devices will be naturally compared to WEB. The comparative efficacy of these devices remains to be determined; therefore, the results of these studies are much anticipated. 

Surface modification represents a promising approach to enhancing the performance of intrasaccular devices. This technique has been effectively utilized in flow diverters (FDs) to mitigate thromboembolic complications and facilitate endothelialization. Zoppo et al. described how surface modification with phosphorylcholine, PMEA, and glycans—as well as with coating with fibrin–heparin and heparin—have decreased thrombogenicity [88]. Although thromboembolic events are more commonly associated with endoluminal FDs, they may also occur with intrasaccular devices. Furthermore, anti-34 antibodies and CD31 analogs covalently bound to FDs and UV irradiation of nitinol have been shown to promote neointimal growth for endothelialization [88]. Given these findings, investigating surface modification aimed at decreasing thrombogenicity and facilitating endothelialization in intrasaccular devices is warranted. 

The use of dual-layer versus single-layer devices regarding aneurysm occlusion rates should be further explored as well. Newer WEB generations have trended away from double-layer devices (i.e., WEB-DL) to single-layer (i.e., WEB-SL and WEB-SLS) mainly to improve their thrombogenicity and deliverability profile. However, most of the newer intrasaccular devices—such as Artisse/LUNA, Contour, and SEAL—are dual-layered. In a preclinical rabbit elastase model, SEAL devices produced significantly higher complete occlusion rates (80% vs. 21%) and neointimal coverage rates (86 ± 15% vs. 49 ± 27%) compared with WEB-SL [85,89]. These findings are supported by Hecker et al., who showed higher rates of complete occlusion with the double-layered Contour device compared with single-layered WEB-SL and WEB-SLS devices [84]. Future studies should investigate the differences in occlusion rates between dual-layered and single-layered devices while also considering device thrombogenicity. 

With the current armamentarium of endovascular devices, each offering distinct advantages, exploring their combination could enhance clinical outcomes in managing complex aneurysms. White et al. documented their experiences of combining WEB with FD for the treatment of eight aneurysms in seven patients. Except for one aneurysm, all had large arterial branches arising from the aneurysm neck. There were no complications, and immediate contrast stagnation in all the aneurysms was seen on immediate post-embolization angiography [90]. Additionally, Diana et al. and Wodarg et al. presented case series on combining Contour with coiling in mostly WNAs [41,91]. Future studies should further investigate the outcomes and risks of combining intrasaccular devices with the more conventional embolization devices, specifically endoluminal remodeling or adjuvant coiling. 

Given the ever-expanding range of endovascular devices, there is a need to develop methods for appropriate device sizing and selection. Each intrasaccular device requires specific sizing considerations. For example, Shah et al. utilized 3D rotational angiography and auto-segmentation to demonstrate a strong correlation between aneurysm volumes and WEB volumes, which may aid in appropriate size selection [92]. Similarly, Ansari et al. found that the highest rate of successful WEB implantation occurred when the device-to-aneurysm volume ratio yielded a value between 0.6 and 0.8 [93]. More data to optimize sizing will be important. Furthermore, the use of artificial intelligence to aid in device sizing and other aspects of decision-making for device selection is an area of significant potential. Jadhav et al. already introduced a machine learning model to aid in predicting the outcome of treatment with a WEB device for WNBAs [94]. Future studies should continue incorporating artificial intelligence and technology to optimize the selection of device(s) for aneurysm treatment.

## 4. Conclusions

The more conventional endovascular techniques, such as BAC, SAC, and FD, have their drawbacks when treating WNBAs. Intrasaccular devices including WEB, the Artisse Embolization System, Contour, and SEAL focus on the aneurysm neck and represent a recent revolution in the treatment of WNBAs. WEB is currently the only intrasaccular device with FDA approval. The completion of more studies evaluating the Artisse Embolization Device, Contour, and SEAL is much anticipated. Future studies should further investigate surface modification, dual-layered vs. single-layered intrasaccular devices, and the combination of intrasaccular devices with more conventional embolization devices, as well as the incorporation of artificial intelligence and other technologies to optimize device sizing and selection.

## Figures and Tables

**Table 1:** Device Comparison.

Device	Shape	Method of Delivery
WEB-SL/SLS	Cylindrical (WEB-SL)	0.017- to 0.033-inch microcatheter
	Spherical (WEB-SLS)	
Artisse/LUNA	Spheroid or flared acorn-like (Artisse)	0.021-inch microcatheter
	Ovoid (LUNA)	
MED	Braided filaments oriented along loops	0.021-inch microcatheter
Contour	Bowl-shaped hemi-sphere	0.017- to 0.027-inch microcatheter
SEAL	Base bridging component ± ovoid upper loop	0.027-inch microcatheter
Trenza	Omega-shaped basket construct that is filled with coils	Excelsior 1018 (Stryker, Kalamazoo, MI, USA) microcatheter

**Table 2:** Important studies of intrasaccular devices.

Device	Name of Study	Year	Authors
WEB	WEB Treatment of Intracranial Aneurysms: Clinical and Anatomic Results in the French Observatory	2016	Pierot et al. [55]
WEB	Safety and efficacy of aneurysm treatment with WEB: results of the WEBCAST study	2016	Pierot et al. [56]
WEB	Safety and Efficacy of Aneurysm Treatment with the WEB: Results of the WEBCAST 2 Study	2017	Pierot et al. [57]
WEB	Safety and efficacy of aneurysm treatment with WEB in the cumulative population of three prospective, multicenter series	2018	Pierot et al. [58]
WEB	The safety and effectiveness of the Woven EndoBridge (WEB) system for the treatment of wide-necked bifurcation aneurysms: final 12-month results of the pivotal WEB Intrasaccular Therapy (WEB-IT) Study	2019	Arthur et al. [60]
WEB	Aneurysm treatment with the Woven EndoBridge (WEB) device in the combined population of two prospective, multicenter series: 5-year follow-up	2023	Pierot et al. [59]
WEB	Safety and effectiveness of the Woven EndoBridge (WEB) system for the treatment of wide necked bifurcation aneurysms: final 5 year results of the pivotal WEB Intra-saccular Therapy study (WEB-IT)	2023	Fiorella et al. [61]
WEB	CLinical Assessment of WEB device in Ruptured aneurYSms (CLARYS): 12-month angiographic results of a multicenter study	2023	Spelle et al. [67]
WEB	CLinical EValuation of WEB 17 device in intracranial aneuRysms (CLEVER): 1-year effectiveness results for ruptured and unruptured aneurysms.	2024	Spelle et al. [52]
LUNA/Artisse	The LUNA aneurysm embolization system for intracranial aneurysm treatment: short-term, mid-term and long-term clinical and angiographic results	2018	Piotin et al. [73]
LUNA/Artisse	The ARTISSE intrasaccular device for intracranial aneurysm treatment: short-term, mid-term and long-term clinical and angiographic results	2022	Piotin et al. [74]
MED	The Medina Embolic Device: early clinical experience from a single center	2017	Aguilar Perez et al. [78]
MED	Medina^®^ Embolization Device for the Treatment of Intracranial Aneurysms: Safety and Angiographic Effectiveness at 6 Months. Neurosurgery	2018	Sourour et al. [77]
MED	The Medina Embolic Device: Karolinska experience	2018	Bhogal et al. [80]
MED	Medina embolization device for the treatment of intracranial aneurysms: 18 months’ angiographic results.	2019	Haffaf et al. [79]
Contour	Endovascular treatment of wide-necked intracranial aneurysms using the novel Contour Neurovascular System: a single-center safety and feasibility study	2020	Akhunbay-Fudge et al. [46]
Contour	The Safety and Effectiveness of the Contour Neurovascular System (Contour) for the Treatment of Bifurcation Aneurysms: The CERUS Study	2022	Liebig et al. [82]
Contour WEB	Comparison of the Contour Neurovascular System and Woven EndoBridge device for treatment of wide-necked cerebral aneurysms at a bifurcation or sidewall	2022	Hecker et al. [84]
Contour	Endosaccular flow disruption with the Contour Neurovascular System: angiographic and clinical results in a single-center study of 60 unruptured intracranial aneurysms	2023	Biondi et al. [81]
Contour	Contour Neurovascular System for endovascular embolization of cerebral aneurysms: a multicenter cohort study of 10 European neurovascular centers	2024	Griessenauer et al. [83]
SEAL	Treatment of a ruptured shallow trilobed cerebral aneurysm with the novel saccular endovascular aneurysm lattice (SEAL) device: A case report with one year follow-up	2023	Pabon et al. [47]
Trenza	Initial Experiences with the Trenza Embolization Device for the Treatment of Wide-Neck Intracranial Aneurysms: A 12-Patient Case Series	2024	Raj et al. [87]

## Data Availability

Not applicable.
